# MatKG: An autonomously generated knowledge graph in Material Science

**DOI:** 10.1038/s41597-024-03039-z

**Published:** 2024-02-17

**Authors:** Vineeth Venugopal, Elsa Olivetti

**Affiliations:** Massachusetts Institute of Technology (MIT), Department of Material Science and Engineering, Boston, 02139 USA

**Keywords:** Computational methods, Design, synthesis and processing

## Abstract

In this paper, we present MatKG, a knowledge graph in materials science that offers a repository of entities and relationships extracted from scientific literature. Using advanced natural language processing techniques, MatKG includes an array of entities, including materials, properties, applications, characterization and synthesis methods, descriptors, and symmetry phase labels. The graph is formulated based on statistical metrics, encompassing over 70,000 entities and 5.4 million unique triples. To enhance accessibility and utility, we have serialized MatKG in both CSV and RDF formats and made these, along with the code base, available to the research community. As the largest knowledge graph in materials science to date, MatKG provides structured organization of domain-specific data. Its deployment holds promise for various applications, including material discovery, recommendation systems, and advanced analytics.

## Background & Summary

In science, few fields offer as much wealth and complexity as materials.Yet, this knowledge is distributed across millions of scientific papers, databases, and other sources, making it challenging to integrate and exploit effectively. It is estimated that at least 10 million scientific papers are related to materials science^1^, covering a diverse range of topics such as material synthesis and processing, materials characterization techniques, mechanical, electrical, magnetic, optical, and photonic properties of materials, thermodynamic and transport properties, materials for energy and environmental applications, biomaterials and biomedical applications, nanomaterials and nanotechnology, composites and hybrid materials, sustainable materials and green chemistry, materials for electronic, optoelectronic, and spintronic devices, materials for advanced manufacturing and 3D printing, materials for aerospace and transportation applications, and computational materials science and materials informatics. Furthermore, this corpus grows at the rate of about a million papers a year at present^1^.

The assimilation of this corpus for current and future scientific research has become a challenge for all scientific and technological advancements leading to increased siloing of information within topical subcommunities^2^, restricted design and exploration spaces of materials, and the absence of machine readable property-chemistry-processing databases^3,4^. The latter specifically could be one of the reasons why AI-driven materials discovery lags behind^5^ other fields such as vision^6^, natural language processing^7^, and robotics^8^

The apparent dissonance and lack of structure in databases make querying materials data a difficult and often frustrating task^2,9^. For example, it is challenging to answer generic questions such as identifying all the properties of a given material (e.g., “what are the properties of single crystal LiMnO_3_?”), listing all the materials that possess a particular property (e.g., “what are all the materials that are thermoelectric?”), identifying all the properties associated with a given processing method (e.g., “what are all the defects shown by solid state sintered bulk lead zirconium titanate”), or determining whether a particular characterization method has been attempted on a given material (e.g., “has a high-temperature AFM study been performed on a TiO2-PVDF composite system?”) without consulting a domain expert or relevant literature. In contrast, other knowledge bases such as Wikidata^10^, Google^11^, Bing, Ask.com, DuckDuckGo, etc have become powerful tools for answering specific questions, such as “What is fly ash?” (A fine powdery residue from coal fired plants) or “What is the capital of Tonga?” (Nuku’alofa) without the need to navigate through other web resources.

The lack of a federated and structured materials database remains a significant bottleneck for data-driven discovery in this field. Current databases of scientific literature such as Scopus, Web of Science, and Crossref only index papers by their keywords, making it difficult to extract nuanced data. Furthermore, recent online data repositories such as Materials Project^12^, OQMD^13^, and NOMAD^14^ largely contain data computed through quantum mechanical calculations, which may not necessarily correspond to experimental observations. While these resources provide valuable information, they are not always aligned with the needs of experimentalists or the broader materials science community. A more comprehensive and integrated approach to organizing and sharing materials data is needed to enable effective data-driven discovery and to advance the field.

One promising solution is the use of Knowledge graphs, which can represent data as a network of interconnected entities and relationships^15^, enabling researchers to navigate and explore data more efficiently. Knowledge graphs provide a powerful solution for representing and integrating heterogeneous data from multiple sources, including scientific papers, databases, and ontologies. By mapping out relationships between entities, knowledge graphs enable researchers to connect and analyze data in new ways, facilitating data-driven discovery in materials science. They also offer a means of organizing and sharing data in a more comprehensive and accessible way, ultimately accelerating scientific progress and advancing our understanding of the world around us.

Knowledge graphs (KGs) are currently being used in a wide range of fields and applications, including search engines^16^, social media platforms^17^, recommendation systems^18^, healthcare^19^, finance^20^, and more^21^. In materials science, several knowledge graphs have been developed to integrate and organize data from various sources, including scientific papers, databases, and ontologies, to support data-driven research and discovery. Examples include the Materials Experiment Knowledge Graph^22^, the Materials Platform for Data Science (MPDS)^23^, Propnet^24^, and the Open Organic Materials Database^25^, among others. These knowledge graphs are being used to advance materials science research, from developing new materials to optimizing existing ones, and are helping to pave the way for more efficient and effective data-driven discovery in the field.

In addition, some domain specific knowledge graphs and knowledge organization schemes such as mof-kg^26^ and Nanomine^27^ have also been proposed. However, these knowledge graphs require manual curation of data and the development of custom ontologies which are both laborious and time consuming. Consequently, they have largely been limited in scope and contain relatively few entities and relationships when compared to the controlled vocabulary of the domain they serve.

In this paper, we introduce MatKG, an extensive knowledge graph of materials science that captures a diverse range of entities and relationships from literature. MatKG includes materials, properties, applications, characterization methods, synthesis methods, symmetry phase labels, and descriptors, among other entities, which are extracted automatically using advanced natural language processing methods. Relationships between these entities are established using statistical metrics, resulting in a knowledge graph with over 70,000 entities and 5.4 million unique triples. MatKG is the largest knowledge graph in the field of materials science at the time of writing, and its development represents a significant advance in the organization and accessibility of materials science data.

## Methods

### Data collection and parsing

A corpus of 5 million scientific papers related to materials science were parsed using Python-based parsers to extract raw text from HTML/XML pages. The detailed development of this database has been described elsewhere^28^ and the code base to reproduce the database is given in the corresponding Github repository^29^. The Elsevier API was used to extract around 20 million image captions from Elsevier publications.

#### Named entity recognition (NER)

The data extraction task was focused solely on the abstracts and figure captions within the corpus. Due to the nature of the role that they serve within the paper, these sections tend to be highly focused and contain very little information peripheral to the main hypothesis of the study^2,3^. This is relevant for the subsequent relation determination task which relies on a statistical count of triples and hence can be easily biased by noise from other sections.

A BERT-based NER model was used to classify every token in the document (i.e., abstract or figure caption) into one of seven categories: Materials (CHM), Symmetry Phase Label (SPL), Synthesis Method (SMT), Descriptor (DSC), Property (PRO), Characterization Method (CMT), and Application (APL)^30^. It is well known that BERT models perform best when their domain of application is concurrent with its domain of training and hence a MatBERT model^31^ - trained on a corpus of some 5 million papers on Material Science - was used as the base model. The specific NER schema and the performance of MatBERT on a test set have been evaluated elsewhere^32^.

Each extracted entity, its NER tag, part of text (abstract, caption) and the DOI of the paper from which it is extracted are stored as triples in the form [Entity, Tag, Part-of-text, DOI], resulting in a list of over 85 million triples.

#### Data cleaning

The raw strings extracted by the transformer-based NER model are subject to a variety of aberrations, enumerated as follows:

1. **Syntactic Variations**: These encompass variations stemming from grammatical idiosyncrasies and diverse punctuation or notation. Examples include:
  [‘electrodes.’, ‘electrodes,’, ‘Electrode’, ‘electrodes:’, ‘electrodes;’, ‘Electrodes’, ‘electrodes)’, ‘Electrodes:’, “electrode’s”, “electrodes”], [‘nano-hybrid’, ‘nano-hybrids’, ‘nanohybrid’, ‘nanohybrids’], [‘Microtwins’, ‘micro-twins’]
2. **Semantic Variations**: These refer to phrases with synonymous vocabulary but varied linguistic expressions. For instance: [‘Dry Reforming Of Methane’, ‘Dry Reforming Of Methane Reaction’], [‘Light-Harvesting Ability’, ‘Light-Harvesting Capability’]
3. **Non-ASCII Entities**: These consist of special characters such as Greek letters and mathematical symbols.
4. **Equivalent Entities**: These phrases represent the same concept but utilize different vocabularies. Examples are: [‘CH4’, ‘Methane’], [‘NH3’, ‘Ammonia’], [‘SEM’, ‘Scanning Electron Microscope’]

In the current iteration of MatKG, equivalent entities remain unmodified. However, the graph-based formalism lends itself well to downstream applications for identifying semantically similar entities.

To disambiguate and standardize other entities, we employ the following methodology:

1. Entries containing purely non-ASCII characters, such as Greek letters, are purged from the database. This helps to maintain data uniformity and simplify processing, especially given the difficulty in distinguishing symbols denoting properties from those used as variables in equations.
2. The remaining entities are sorted and clustered based on their Levenshtein edit distance using Python’s Fuzzy Sort algorithm. This effectively groups similar entities, for instance, [‘electrode’, ‘electrodes’], based on stringent thresholds of 95% and 90% fuzzy similarity. However, this approach may inadvertently group semantically dissimilar entities, such as [‘methanol’, ‘ethanol’] and [‘chemical age’, ‘chemical image’].
3. Subsequently, the ChatGPT API is employed to deduce a canonical representation for each cluster of similar entities. For example, it returns ‘electrode’ as the canonical form for both ‘electrode’ and ‘electrodes’, while retaining disparate entities like ‘chemical age’ and ‘chemical image’ as distinct. The performance of ChatGPT on identifying chemical strings was specifically improved by using few-shot prompts that included numerous examples of similar chemical terms, thereby enhancing its ability to recognize and differentiate between such terms accurately.
4. The canonical entity identified in step 3 is used to standardize all entities within its respective cluster.
5. This entire sequence of operations is iterated five times.

The use of ChatGPT was found to be significantly helpful in identifying a general English language form of the entities under consideration and is crucial in the automation process. Indeed, recent studies have shown that ChatGPT outperforms crowd work in many tasks^33^. The exact prompt used for the API call is included in the code repository^34^.

#### Relationship determination

MatKG is constructed by merging rows that share identical DOIs. Specifically, consider two entities, *E*1 and *E*2, that are found under the same DOI, termed as DOI-1. If the database houses the triples [*E*1, Tag1, DOI–1] and [*E*2, Tag2, DOI-1], then a novel triple [*E*1, rel, *E*2] is created. In this context, rel is defined as Tag1-Tag2, and a co-occurrence frequency *v* is assigned to this triple, where *v* is the number of DOIs containing both *E*1 and *E*2.

For example, if *Fe*2*O*_3_ with an NER tag of CHM and ‘catalyst’ with an NER tag of APL are discovered to co-occur in 123 documents, then the triple [Fe_2_*O*_3_, CHM-APL, ‘catalyst’] is added to MatKG. Furthermore, a weight factor of 123 is affixed, thus forming the quartet [Fe_2_*O*_3_, CHM-APL, ‘catalyst’, 123]. Semantically, this signifies a strong material-application association between *Fe*2*O*_3_ and ‘catalyst’. Additional analyses from neighboring nodes enable us to deduce that ‘catalyst’ is indeed an application for *Fe*2*O*_3_, particularly in the decomposition of sulfuric acid^35^. The intricacies of such connections will be further elucidated in the section on technical validation.

#### Linking to other databases

The integration of external knowledge sources is an essential feature of knowledge graphs. Wikidata/DBpedia is a large-scale, open, and linked data knowledge base that serves as a central hub for the semantic web^10^. As such, it represents an invaluable resource for knowledge graph construction, especially in areas such as material science where there is a high degree of interdisciplinarity. Therefore, the entities in MatKG were searched within Wikidata and the corresponding URLs were recorded.

The outcome of this search is twofold. First, it enables the enrichment of MatKG with additional information from Wikidata. For example, if a material entity in MatKG is linked to a chemical compound entity in Wikidata, additional information about the compound such as its molecular weight or boiling point can be added to MatKG. Second, it provides a mechanism for linking MatKG to other knowledge graphs and datasets that are already represented in Wikidata. This is important for enabling cross-domain knowledge discovery and integration.

The process of searching for MatKG entities within Wikidata was performed using a Python script that queried the Wikidata API for each entity in the MatKG. The script utilized the Levenshtein distance algorithm to match the entity names in MatKG with those in Wikidata, allowing for the identification of potential matches with slight variations in spelling or formatting. The API returned a list of URLs corresponding to potential matches in Wikidata, which were then recorded and stored in MatKG as part of the entity metadata.

The inclusion of Wikidata URLs in the MatKG entity metadata provides additional contextual information and connections to external knowledge sources, allowing for more comprehensive and diverse data analysis. In cases where multiple URLs were returned for a given entity, the user can choose the one that is most relevant for their specific research task. The inclusion of Wikidata links also facilitates the integration of MatKG with other knowledge graphs and databases, enabling cross-disciplinary research and collaboration.

The SparkQL API of wikidata identified around 53740 entities that were found to have endpoint URL, representing 61 percent of all entities in MatKG. Additionally, the PyMatGen rest API was used to link 3000 chemical entities in MatKG with material records in the Materials Project^12^.

## Data Records

Knowledge graphs are frequently represented using specialized database languages, including but not limited to the Resource Description Framework (RDF)^36^, Labeled Property Graphs (LPG)^37^, Web Ontology Language (OWL)^38^, and JSON-LD^39^. Such formats facilitate the integration of these graphs into the broader semantic web and optimize their accessibility via existing search engines. It is important to note that each of these data formats have its own set of merits and limitations, making the choice of format contingent upon the specific objectives and constraints of the project. In the case of MatKG, the knowledge graph is articulated as an amalgamation of two distinct RDF graphs^40^, details of which are delineated in subsequent sections. For direct access to the raw data, we additionally provide the dataset in CSV file format^40^.

The dataset generated through BERT-based Named Entity Recognition (NER) models comprises an extensive collection that associates a ‘raw entity’ with its corresponding attributes: ‘NER Tag’, ‘Part of Text’, ‘Digital Object Identifier (DOI)’, and ‘Preferred Entity’. The term ‘raw entity’ denotes the string as initially extracted, prior to any data cleaning procedures described in the Methods section. Following this cleaning process, the resultant form of the raw entity is designated as the ‘Preferred Entity’ and is included with each dataset entry. It should be noted that raw entries discarded during the cleaning stage are labeled with ‘None’ as their Preferred Entity. Additionally, to ensure a comprehensive record, the ‘Year’ corresponding to the publication of each DOI is appended to the dataset. The ‘NER Tag’ corresponds to one of the seven ontological categories, namely ‘CHM’, ‘PRO’, ‘APL’, ‘SMT’, ‘CMT’, ‘DSC’, and ‘SPL’. The ‘Part of Text’ field indicates the source of the raw entity, distinguishing between the abstract (‘abs’) and the caption (‘cap’) of the document. This dataset, containing in excess of 85 million entries, serves as an exhaustive catalog of ontological categories in Materials Science, complete with their respective mappings. The collection is disseminated in the form of a CSV file, denominated as ‘ENTPTNERDOI’.

The [subject, relationship, objec, count] dataset is generated from ENTPTNERDOI through the procedure described in Methods. This dataset has 5.7 million entries as is denominated as SUBRELOBJ in the csv format.

### Resource description framework database

A Resource Description Framework (RDF) database is a type of database that uses a standardized format for representing and exchanging information. RDF databases are particularly relevant for knowledge graphs because they enable the representation of data as a set of triples consisting of a subject, a predicate, and an object^36^. This structure enables the representation of complex relationships between entities, facilitating efficient and flexible querying of the data. The subject is the resource being described, the predicate is the relationship between the subject and the object, and the object is the value of the relationship. The subject, predicate, and object are typically represented as URIs (roughly equivalent to a URL) or literals in RDF data^41^.

RDF databases can be queried using a standardized query language called SPARQL (SPARQL Protocol and RDF Query Language), which allows for complex queries across multiple triples and graphs^42^. SPARQL queries can be used to retrieve specific subsets of data from the RDF database, enabling researchers to extract meaningful insights and patterns from the data. An example SPARQL query to retrieve the properties of graphene from MatKG is given in Table 1.

In the case of MatKG, we have created an RDF database using the python library RDFLib^43^ to enable efficient storage, retrieval, and querying of the vast amount of data captured in the knowledge graph. By representing MatKG as an RDF database, we are able to take advantage of the standard format and query language of RDF, as well as the many tools and technologies that have been developed to work with RDF data. This enables us to more easily integrate MatKG with other RDF-based knowledge graphs and to leverage existing RDF-based tools and frameworks for data analysis and visualization.

**Fig. 1:**
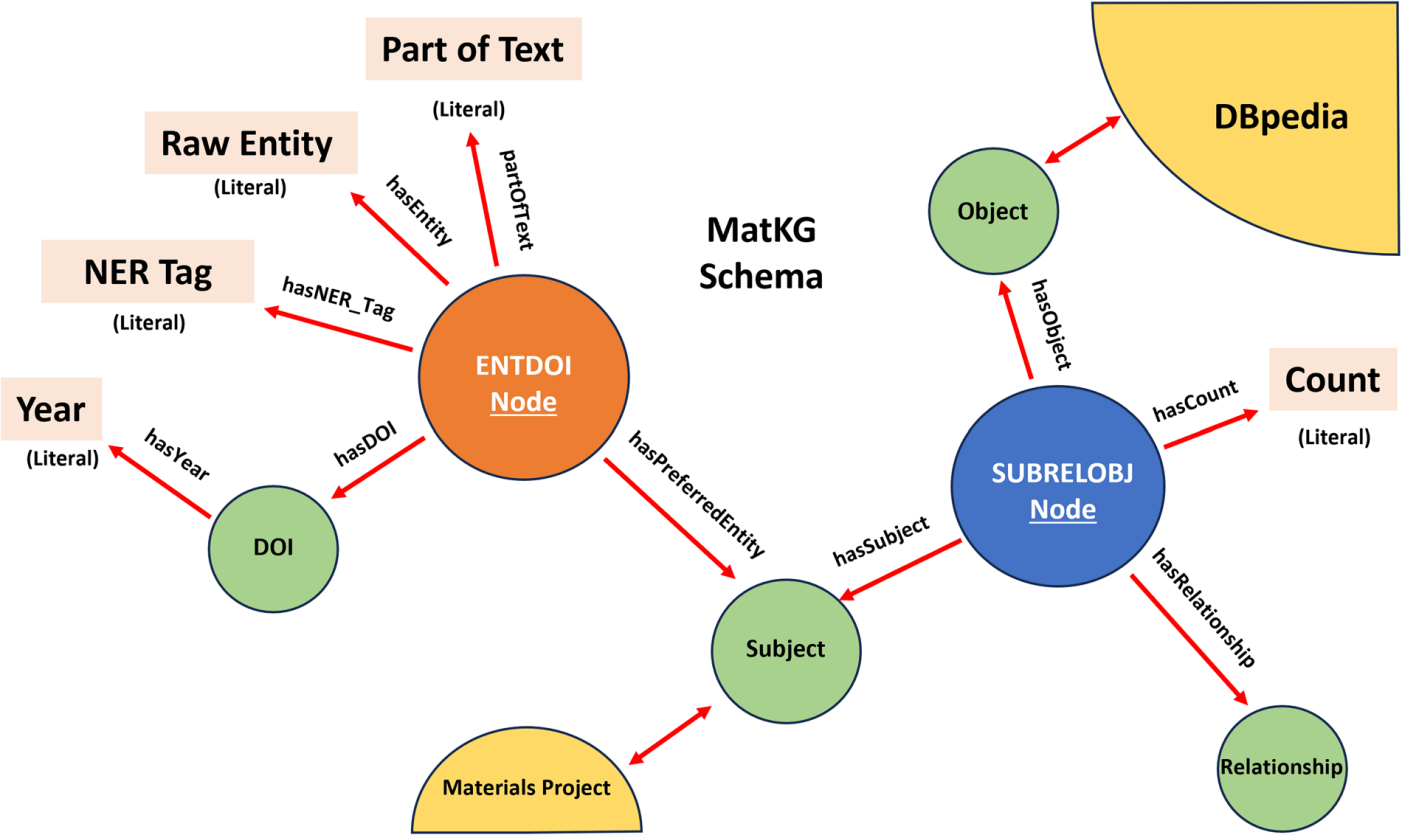
This schematic represents the MatKG RDF dataset schema, mapping out the interconnections between its elements. The central ‘ENTDOI Node’ links to the ‘Raw Entity’ and ‘NER Tag’ as well the bibliographic data via ‘Year’ and ‘DOI’. It interfaces with the ‘SUBRELOBJ Node’ that branches out to ‘Object’, ‘Subject’, and ‘Relationship’ nodes, demonstrating the dataset’s relational structure. The ‘DBpedia’ and ‘Materials Project’ nodes indicate integration with external data sources and specific data subsets.

Specifically, MatKG is the union of two RDF Graphs, ENTPTNERDOI and SUBRELOBJ. The overall schema is described below and illustrated in Fig. 1

#### ENTPTNERDOI

The [‘raw entity’, ‘part of text’, ‘NER tag’, ‘DOI’, ‘year’] dataset is expressed as ENTPTNERDOI.nt file with the following schema:

- Each row in the dataset serves as a distinct *node* in the ENTPTNERDOI graph.
- The *raw Entity* is denoted as a literal and is associated with its respective node via the predicate hasEntity.
- The *part of text* is denoted as a literal and is linked to the node through the predicate partOfText.
- The *NER Tag* is denoted as a literal and linked to the node by the predicate hasNER_Tag.
- The *DOI* is denoted as a URI and is linked to the node using the predicate hasDOI.
- The *preferred entity* is denoted as a URI and is linked to the node using the predicate hasPreferredEntity.

In addition,

- The *year* is denoted as a literal and is linked to the *DOI* node using the predicate hasYear.

#### SUBRELOBJ

The [subject, relationship, object] triple is expressed as SUBRELOBJ.nt file with the following schema:

- Each row in the dataset acts as a unique *node* in the SUBRELOBJ graph.
- The *subject* is represented as a URI and is associated with its corresponding node through the predicate hasSubject.
- The *object* is also represented as a URI and is linked to its respective node via the predicate hasObject.
- The *relationship* is designated as a URI and is connected to the node using the predicate hasRelationship.
- The *count* is denoted as a literal and is associated with the node through the predicate hasCount.

The two RDF graphs, ENTPTNERDOI and SUBRELOBJ, are interconnected through specific nodes: the ‘preferred entities’ node in ENTPTNERDOI and the ‘subject’ and ‘object’ nodes in SUBRELOBJ. Essentially, the URIs for the preferred entities in the ENTPTNERDOI graph are reused to create the subject and object nodes in the SUBRELOBJ graph. This congruency in URIs serves as a linking mechanism, enabling the association of [subject, relationship, object] triples in the SUBRELOBJ graph with the corresponding DOIs, years of publication, and part-of-text information present in the ENTPTNERDOI graph.

Further, the code base^34^ allows the identification of the most similar indexes from Wikidata/DBpedia and Materials Project to the ‘prefered entity’ URIs, allows crosssection of MatKG with these databases. Mapping to WikiData was chosen due to its comprehensive coverage and open-access nature, which aligns well with our goal of enhancing the accessibility and interconnectedness of materials science data. WikiData’s structured format and widespread use facilitate the integration of diverse data sources, making it an invaluable resource for expanding the reach and utility of MatKG. Similarly, the Materials Project was selected for its extensive database of material properties and its prominence in the materials science community. By linking MatKG with the Materials Project through the PyMatGen rest API^44^, we are able to enrich our knowledge graph with detailed material property data, thereby enhancing the depth and practical applicability of MatKG for researchers and practitioners in the field.

## Technical Validation

As noted earlier, the extracted tokens have gone through several rounds of cleaning, checking, and assimilation from the NER extraction step. During this process tokens that do not meet the requirements of a controlled vocabulary such as incomplete strings (‘-methyl’, ‘-(OH)2’, ‘ reaction’, etc), syntactic and semantic variations of the same string (‘electroded’, ‘electrodes’, ‘reaction’, ‘reactions’, etc) and non-ascii character strings (‘rho’, ‘tau’, ‘phi’) are either removed, altered, or merged with a more suitable morphological representation. During this stage, the total number of extracted entities reduced from around half a million to just under 70,000 while the number of extracted triples diminish from 11 million to 5.4 million, suggesting that the cleaning process is able to concentrate the triple database.

For further validation, 1500 entities from the database were randomly selected and checked by five human annotators who were graduate students in Material Science. The entities were selected such that each annotator received 70% entities that had a high degree in the graph (>1000) while 30% had a low degree (<100). Since nodes with high degrees are present in a larger number of triples, ultimately this ensured that around 200,000 rows were checked for morphological conformation and validity.

The annotators were presented with the NER tag of the entity and were asked to report if they agreed with the tag assigned to the entity by the MatBERT-NER model. The results of this experiment are presented in Table 2.

**Table 1:** An Example SPARQL Query to extract the properties of graphene from MatKG.

SPARQL Query
PREFIX ex:<http://example.com/>
SELECT?object?count WHERE
?index ex:hasSubject <http://example.com/CHM/Graphene>;
ex:hasRelationship <http://example.com/CHM-PRO>;
ex:hasObject?object;
ex:hasCount?count.
ORDER BY DESC(?count)

**Table 2:** The annotater agreement/disagreement metrics of the entities in MatKG on a randomly selected test set.

Tag	Number in Test Set	Number disagreed	% Disagreement
Application	74	2	2.7
Material	318	3	0.9
Characterization Method	111	6	5.4
Descriptor	101	6	5.9
Property	517	69	13.3
Synthesis Method	304	67	22.03
Symmetry Phase Label	75	19	25.3

It is observed that for the categories of application, material, characterization method, and descriptor, the level of disagreement between the assigned tag and the model-defined tag is below 6 percent. For the synthesis and symmetry phase label categories, this number is higher but still less than 30% all entities in the category. It is important to note that certain entities may be difficult to strictly categorize as one or the other NER tag, hence such behavior is expected. However, a complete understanding of entities requires the consideration of multiple NER tags to capture the different aspects of the entity’s characteristics. The NER model’s performance is crucial in ensuring the accuracy of the knowledge graph, and the relatively low disagreement percentage indicates its suitability for the task.

Due to the size of MatKG, manually validating each extracted relationship is not feasible. To address this, co-occurrence frequency has been incorporated as a weighted parameter within the tripleset, functioning as an implicit validation method. Essentially, a higher co-occurrence frequency between two entities suggests a stronger correlation and, consequently, higher confidence in their relational association. It’s important to note that these relationships, extracted through statistical methods, can represent either actual causation, as seen in the example [‘TiO2’, ‘CHM-APL’, ‘Coating’], or mere correlation, like in [‘Alkyl Hydroperoxide’, ‘CHM-CHM’, ‘Alkane’]. Currently, MatKG does not differentiate between causative and correlative relationships, as acknowledged in the ‘limitations’ section.

**Fig. 2:**
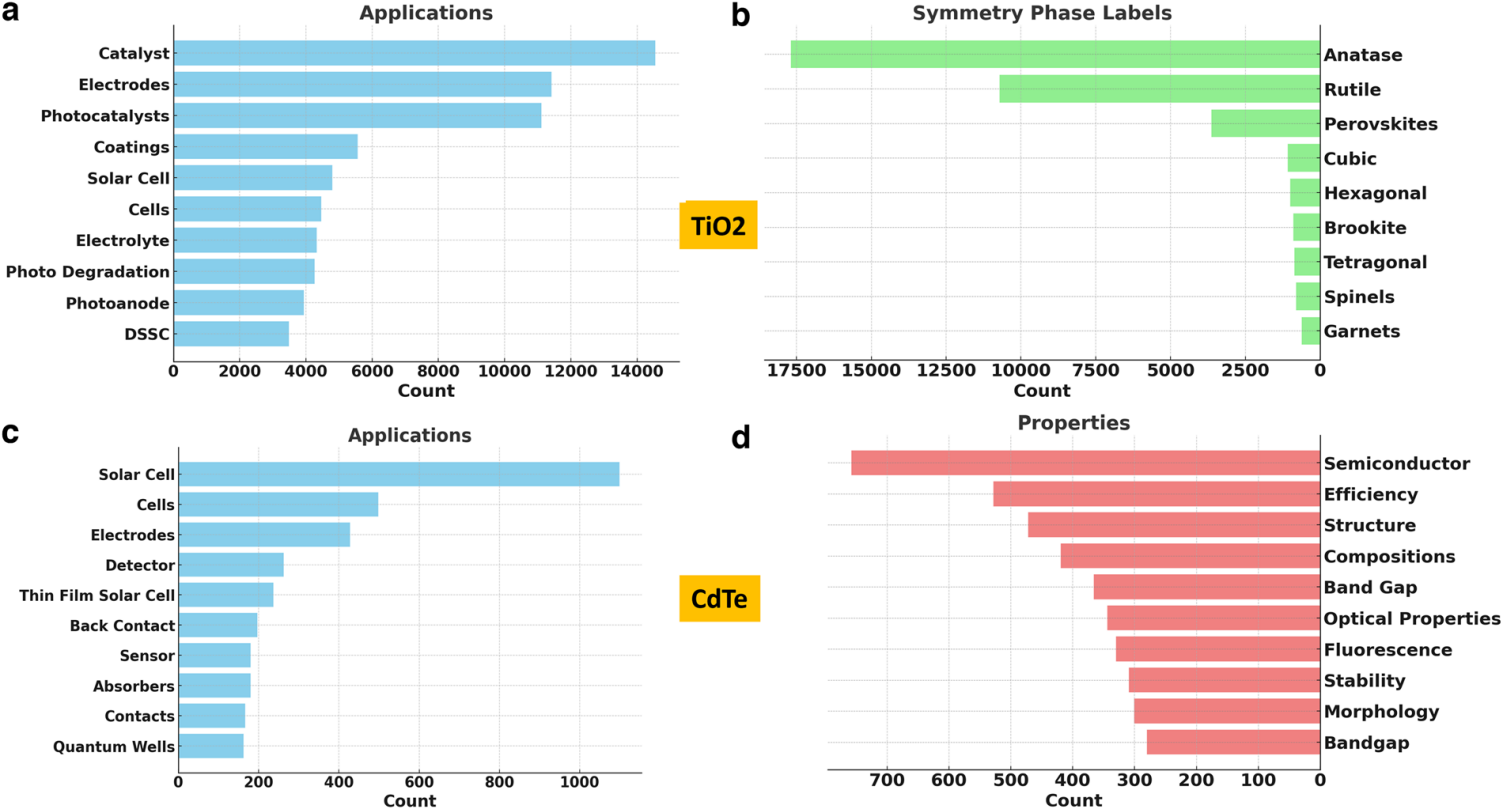
The image shows the top ten applications and properties of TiO2 and CdTe extracted from MatKG. (**a**–**c**) shows that the most common applications of TiO2 and CdTe are photocatalysts and solar cells respectively (**b**) shows that Anatase and Rutile are the main symmetry phase labels of TiO2 and (**d**) shows that semiconductivity and efficiency are the most commonly mentioned properties of CdTe.

However, even with this limitation, MatKG is a powerful knowledge base to query material science literature as shown in Fig. 2. In order to respond to the customized query “What are the applications of TiO2”, the top ten triples with the subject ‘TiO2’ and the relation ‘material-property' are extracted from MatKG. It is immediately seen that the top applications of TiO2 are as electrodes, for coating, as catalysts, for dye sensitized solar cells, photocatalysts etc. Following the same procedure it is seen that the top symmetry and phase labels associated with TiO2 are anatase, rutile, and perovskite. Similarly, for Cadmium Telluride the top applications are seen to be solar cells, optoelectronic detectors, back contacts, absorbers etc while the top properties are seen to be ‘semiconductor’, ‘optical properties’, ‘bandgap’ etc. These agree with our knowledge of these materials and demonstrate the ease with which MatKG can answer focused and nuanced queries from literature, thereby addressing one of the challenges mentioned in the introduction to this paper.

**Fig. 3:**
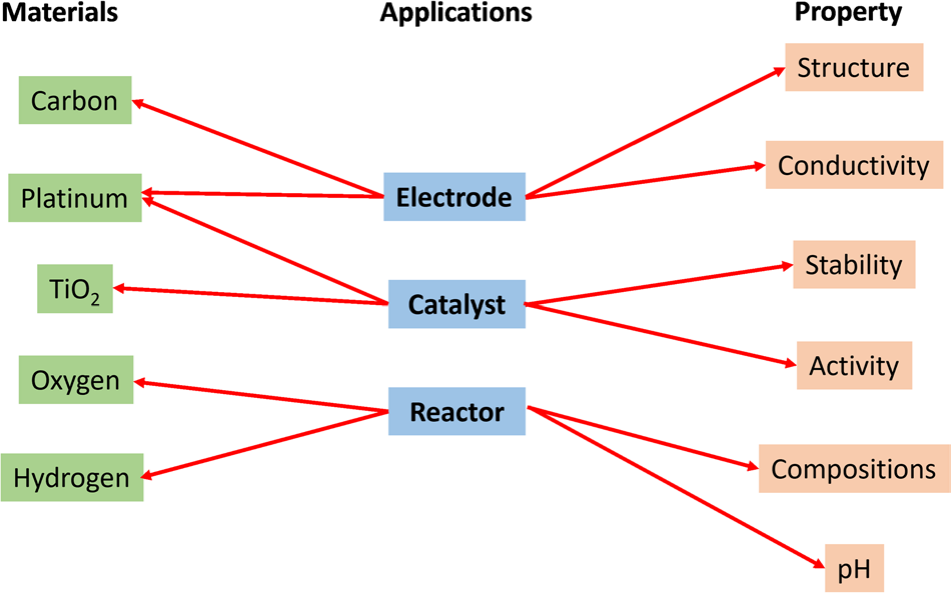
The figure presents bipartite graphs extracted from MatKG, depicting representative relationships between materials and their applications, as well as properties and their applications. For each application, two highly relevant connections are shown.

An extension of this procedure allows for the creation of specific bipartite graphs as shown in Fig. 3 where the top materials and properties associated with some applications are listed. It is seen that the top material associated with being a catalyst is ‘platinum’, which is also strongly associated with electrodes. Similarly, the key property associated with being a catalyst is ‘activity’, while the property associated with electrodes is ‘conductivity’. While these well known relations assert the validity of MatKG as a query and exploration tool, it should be noted that it is very difficult to extract these relations autonomously through other means at this time.

## Limitations

MatKG represents a significant step forward in bringing materials science into the age of the semantic web, both in terms of the breadth of relations it captures and the size of the resulting graph. However, it is important to acknowledge the limitations of the methodology used to generate this knowledge base. Specifically, MatKG captures connections found in the studied corpus, rather than representing the entirety of materials science. With a large corpus of 5 million papers, it is likely that many of the captured relations are asymptotically true while some may not hold up under scrutiny.

While statistical co-occurrence frequency is a useful measure for filtering out noisy correlations, it does not establish causation. For example, a material that is commonly used during a synthesis method and a material synthesized using that method will both have a high co-occurrence frequency for the material-synthesis method triple. However, the former is only a correlation, whereas the latter represents a true causal relationship. Even when there are strong correlations in the extracted triples, their precise relationship can still be different. For example, in MatKG both (‘In2O3’, ‘Optical Material’) and (‘Bismuth’, ‘Nuclear Reactor’) are connected by the relationship ‘CHM-APL’ indicating that the tuple connects a material to an application. However, In2O3 ‘is an’ optical material while Bismuth ‘is used in’ a nuclear reactor. Hence, it is important to make this distinction in the analysis of relations extracted from MatKG. Future versions of the graph can incorporate triples extracted using relationship extraction models that can add high fidelity without significant expression in text, thereby addressing this limitation.

## Usage Notes

The RDF Datasets and the processed CSV files for ENTPTNERDOI and SUBRELOBJ are given in 10.5281/zenodo.10022726^40^.

The github repository^34^ contains detailed tutorial style notebooks that demonstrate:

1. The extraction of the [subject, relationship, object] database from ENTPTNERDOI.csv file
2. The creation of the RDF graphs ENTPTNERDOI.nt and SUBELOBJ.nt from their respective csv files
3. SPARQL based querying of the two RDF graphs to generate Figs. 2, 3 and Table 1.
4. Linking of entities in the RDF graphs to Wikidata and to the Materials Project

The dataset and the code base allows easy reproduction of the data, as well as nuanced focused query of MatKG.

## Data Availability

The code base is made available in^34^ as noted above.
